# Personalized Approaches to Spine Surgery: Innovations and Future Directions

**DOI:** 10.3390/jpm16050249

**Published:** 2026-05-04

**Authors:** Kai-Uwe Lewandrowski

**Affiliations:** 1 Division of Personalized Pain Research and Education, Center for Advanced Spine Care of Southern Arizona, Tucson, AZ 85712, USA; business@tucsonspine.com; 2 Department of Orthopaedic Surgery, Banner University Medical Center, Tucson, AZ 85719, USA; 3 Centre for Musculoskeletal Surgery (CMSC), Chariteplatz 1, 10117 Berlin, Germany; 4 Department of Orthopaedics, Fundacion Universitaria Sanitas, Bogota 111321, Colombia; 5 Department of Orthopedics, Hospital Universitario Gaffree e Guinle, Universidade Federal do Estado do Rio de Janeiro, Rio de Janeiro 20270-004, Brazil

## 1. Introduction

Personalized spine care is increasingly understood as more than the use of custom implants, robotics, navigation, or advanced imaging alone. It reflects a broader clinical paradigm in which diagnosis, operative strategy, reconstruction, perioperative management, digital monitoring, and follow-up are tailored more closely to the individual patient [1]. This shift is driven by the recognition that spinal disorders are heterogeneous in etiology, biomechanics, symptom presentation, comorbidity profile, and biologic response to intervention. As a result, optimal treatment increasingly depends on the integration of structural, functional, biologic, and patient-reported factors [1,2].

The Special Issue *Personalized Approaches to Spine Surgery: Innovations and Future Directions* brings together eight contributions that collectively illustrate how this broader concept is being translated into contemporary spine surgery and spine care. These contributions highlight several major trends, including biologically informed perioperative assessment, tissue-sparing and minimally invasive strategies, patient-specific reconstruction, digital health monitoring, patient-centered outcome evaluation, and objective physiologic stratification. Rather than representing a single technological pathway, the papers in this collection suggest that personalization is emerging as a multidimensional framework with implications across the continuum of care [1,3,4].

## 2. An Overview of Published Articles

### 2.1. Biologic and Perioperative Personalization

One of the central themes of this Special Issue is the growing recognition that personalization must extend beyond anatomy and instrumentation to include biologic variability in the response to surgical trauma. Contribution 1 addresses this concept by reviewing immune, neuroendocrine, and inflammatory pathways activated by surgery and by emphasizing the potential relevance of biomarkers such as interleukin-6 and C-reactive protein in characterizing perioperative stress and recovery. Although the article is framed more broadly as a review of surgical trauma than as a spine-specific clinical study, it meaningfully broadens the scope of personalized spine care by underscoring that patients differ not only in their structural pathology, but also in the systemic biologic processes that may influence healing and postoperative outcomes [1,5].

### 2.2. Tissue-Sparing and Minimally Invasive Strategies

A second major theme is the continued evolution of techniques toward less disruptive and more targeted interventions. Contribution 2 presents a tissue-sparing posterior cervical fusion strategy designed to reduce soft tissue disruption while preserving the goals of posterior cervical arthrodesis. Importantly, this paper is not only technical in focus; it also emphasizes coding, reimbursement, and market-access considerations, thereby highlighting that successful implementation of personalized surgical solutions depends not only on technical feasibility, but also on practical pathways for clinical adoption.

Contribution 4 further develops this theme by reviewing minimally invasive approaches to spinal cerebrospinal fluid leak repair and by presenting an illustrative case that used flexible endoscopy with a wearable heads-up display. The paper is best understood as a perspective with a novel operative illustration rather than as definitive validation of an established technique. Even so, it usefully shows how personalization may also involve tailoring visualization, access, and intraoperative workflow to difficult clinical scenarios. Together, contributions 2 and 4 suggest that personalized spine surgery increasingly involves selecting the least disruptive approach capable of addressing the patient’s specific pathology [6].

### 2.3. Patient-Specific Reconstruction and Additive Manufacturing

Patient-specific reconstruction represents another important domain in the personalization of spine surgery. Contribution 3 reviews the clinical application of 3D-printed artificial vertebral bodies and highlights the promise of additive manufacturing for improving implant conformity, reducing subsidence, and facilitating osseointegration. At the same time, the review appropriately notes the continued limitations of the evidence base, including study heterogeneity, limited randomized data, manufacturing time, cost, and regulatory barriers. This balanced perspective is important because it places innovation within the realities of translation to clinical practice [3].

Contribution 5 complements this broader reconstructive perspective with stronger clinical data. In a prospective, multi-surgeon, multi-center study of 3D-printed titanium patient-specific interbody cages used in lumbar interbody fusion, the authors report significant improvements in pain and health-related quality of life through 24 months, along with a low reoperation rate and no revisions of the patient-specific cages themselves. Considered together, contributions 3 and 5 indicate that additive manufacturing is evolving from conceptual promise toward clinically meaningful applications in appropriately selected patients [3].

### 2.4. Digital Monitoring and Patient-Centered Outcomes

Personalization in spine care also depends on the ability to monitor patient experience and treatment adherence more continuously over time. Contribution 6 addresses this issue through a mobile health platform designed to track brace wear, discomfort, emotional distress, and social challenges in adolescents with idiopathic scoliosis. The study is best interpreted as a feasibility and clinical-utility investigation rather than as definitive proof of efficacy. Nevertheless, it suggests that app-based monitoring may support the early identification of barriers to treatment adherence and may help clinicians respond more quickly to psychosocial as well as physical concerns [4,7].

A related dimension of personalization is the need to evaluate treatment success through outcomes that matter to patients and families, not solely through radiographic or technical measures. Contribution 7 examines health-related quality of life in cerebral palsy patients with early-onset scoliosis treated with growth-friendly implants. The study reports small, largely non-significant improvements overall, while also suggesting that traditional implants may offer a modest additional benefit over magnetically controlled growing rods in some domains. Although the improvements are appropriately interpreted with caution, this contribution reinforces a broader principle of personalized care: procedural success must ultimately be interpreted in the context of functional benefit and quality of life [2].

### 2.5. Objective Physiologic Stratification and Data-Guided Surgery

Another emerging direction in personalized spine surgery is the use of objective physiologic information to inform intraoperative decision-making. Contribution 8 explores threshold-anchored mechanomyography metrics for patient stratification during spinal decompression and examines their association with early pain outcomes. As an exploratory pilot study, the paper does not establish a validated clinical standard; rather, it introduces a promising framework for measuring decompression adequacy in a more individualized and physiologically grounded manner. If validated in larger cohorts, such approaches could help move intraoperative assessment beyond purely visual or anatomical criteria and toward patient-specific functional interpretation [5,8].

## 3. Conclusions

Collectively, the contributions to this Special Issue show that personalized approaches in spine surgery and spine care are not defined by any single device, technique, or platform. Instead, personalization is emerging as a multilevel framework that integrates biologic understanding, patient-specific reconstruction, less invasive access strategies, digital monitoring, patient-centered outcomes, and objective physiologic assessment [1,2,3,4,6,8].

A central insight from this collection is that personalization must extend beyond technical precision alone. Advances in implant design, imaging, visualization, and monitoring are important, but their value ultimately depends on whether they improve recovery, function, quality of life, and the alignment of treatment with individual patient needs [1,2,4]. At the same time, the expanding availability of biologic, physiologic, and digital data offers new opportunities to refine decision-making and tailor treatment more effectively [1,4,5,8].

Future progress will depend on rigorous validation of emerging technologies, improved integration of biologic and clinical data, and sustained emphasis on patient-centered outcomes [1,2,3,6]. Equally important will be the development of implementation pathways that make these innovations scalable and accessible in daily practice. Ultimately, personalized approaches in spine surgery and spine care will be defined not only by how precisely procedures are performed, but by how effectively treatment and follow-up are adapted to the individual patient [1].
